# Arthroscopic Repair of Labrum Using the Inverted V‐Shape Double‐Strand Vertical Mattress Suture Technique in Hip Joint

**DOI:** 10.1002/atn2.70161

**Published:** 2026-07-25

**Authors:** Yang He, Qi Jia, Lin He, Long Wang, Ming‐xin Wang, Chun‐bao Li

**Affiliations:** ^1^ Department of Orthopedics The Fourth Medical Center of Chinese PLA General Hospital Beijing China

## Abstract

The labral suture is essential in the arthroscopic treatment of hip acetabular labral injuries and maintains its suction sealing effect. The traditional single‐strand loop fixation, with its loop around the labrum tissue, may cause labral eversion and disrupt the sealing effect. Traditional single‐strand vertical mattress suture technique has the advantage of preserving the free edge of the labrum and restoring its suction sealing function. However, for the large labrum tear, the strength of the single‐strand suture may be insufficient for stability. At the same time, more anchors will be needed for the procedure, which is one of the key consumables in arthroscopic surgery. Here, we describe an inverted V‐shape double‐strand vertical mattress suture technique, which preserves the labrum sealing effect by using vertical mattress suture and reduces the number of anchors needed by utilizing a double‐strand anchor and the V‐shape suture.

## Graphical abstract

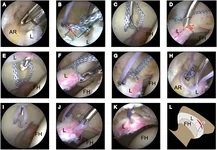

**VIDEO 1:** This video shows a case of right hip labral repair in a supine position, viewing through an anterolateral portal. First, a double‐strand anchor is placed in the acetabular bed at the back of the labral injury 1 to 2 mm from the edge of the labrum. The end of 1 strand is grasped with a suture hook. Then, the suture hook passes through the torn labrum at the labrum and cartilage junction 1 to 2 mm medially from the anchor and introduces the suture into the central compartment. The free suture hook is then passed from near the edge of the labrum, approximately at the outer third. The surgeon grasps the end of the suture from the central compartment to the back of the labrum and knots it in place to hold it in position. Then, the other strand is grasped and displaced 1 to 2 mm laterally from the injured labrum on the other side. Similarly, the suture hook passes through the torn labrum and introduces the suture into the central compartment. The suture hook is passed from near the edge of the labrum, and then, the suture is withdrawn, and the surgeon knots it to hold in place. Video content can be viewed at https://doi.org/10.1002/atn2.70161.

Labral injuries were first identified by Peterson in 1957 in patients with posterior dislocation of the hip^1^ and diagnosed under arthroscopy in 1986 by Suzuki et al.^2^ Hip arthroscopy has gradually replaced open surgery as the first choice for treatment of labral injuries. The commonly used methods include labral debridement, labral repair, and labral reconstruction. Labral suturing is preferred when the quality of the labral tissue is sufficient to restore the “sealing effect.”^3^, ^4^ The traditional single‐strand loop fixation, with its loop around the labrum tissue, may cause labral eversion and disrupt the sealing effect. Traditional single‐strand vertical mattress suture technique can preserve the free edge of the labrum in contact with the femoral head and allow the labrum to perform its suction sealing function.^5^ However, the strength of the single strand may be insufficient, and stability is decreased; therefore, anchors are used in larger quantities. Anchors are one of the key consumables in arthroscopic surgery, and their costs may impact health care costs and the patient's financial burden.^6^ In double‐strand suture technique with loop fixation, the 2 strands often align together when tension is applied. This can compromise blood supply and impair tissue healing, particularly in cases involving hypertrophic or difficult‐to‐repair labrums.

Therefore, optimizing the strategy of anchor usage to achieve a reliable suture result and reduce the number of anchors has become a challenge in the treatment of labral injuries. Here, we describe an inverted V‐shape double‐strand vertical mattress suture technique, which preserves the labrum sealing effect and reduces the number of anchors.

## SURGICAL TECHNIQUE

The patient is placed under general anesthesia, the lower limb is placed in the supine position in the boot of the traction bed (Orthopedic Systems), and the affected limb is fixed in traction with postless distraction technique.^7^ The operation field is routinely disinfected, and sterile sheets are laid. The synovium, ligaments, femoral head and neck junction area, cartilage, acetabulum, and labrum are first explored. Arthroscopic surgery blades (Smith & Nephew, USA) and radiofrequency (Smith & Nephew, USA) are used to clean up the proliferated synovium and trim the damaged portion of the round ligament. The extent of labral injury, as per the clockface method, is explored. The labral tear edges are trimmed with radiofrequency; the acetabular rim is ground to remove the osteophytes, and the anterosuperior acetabular wound is trimmed and freshened with a planer (Smith & Nephew, USA).

**FIGURE 1:**
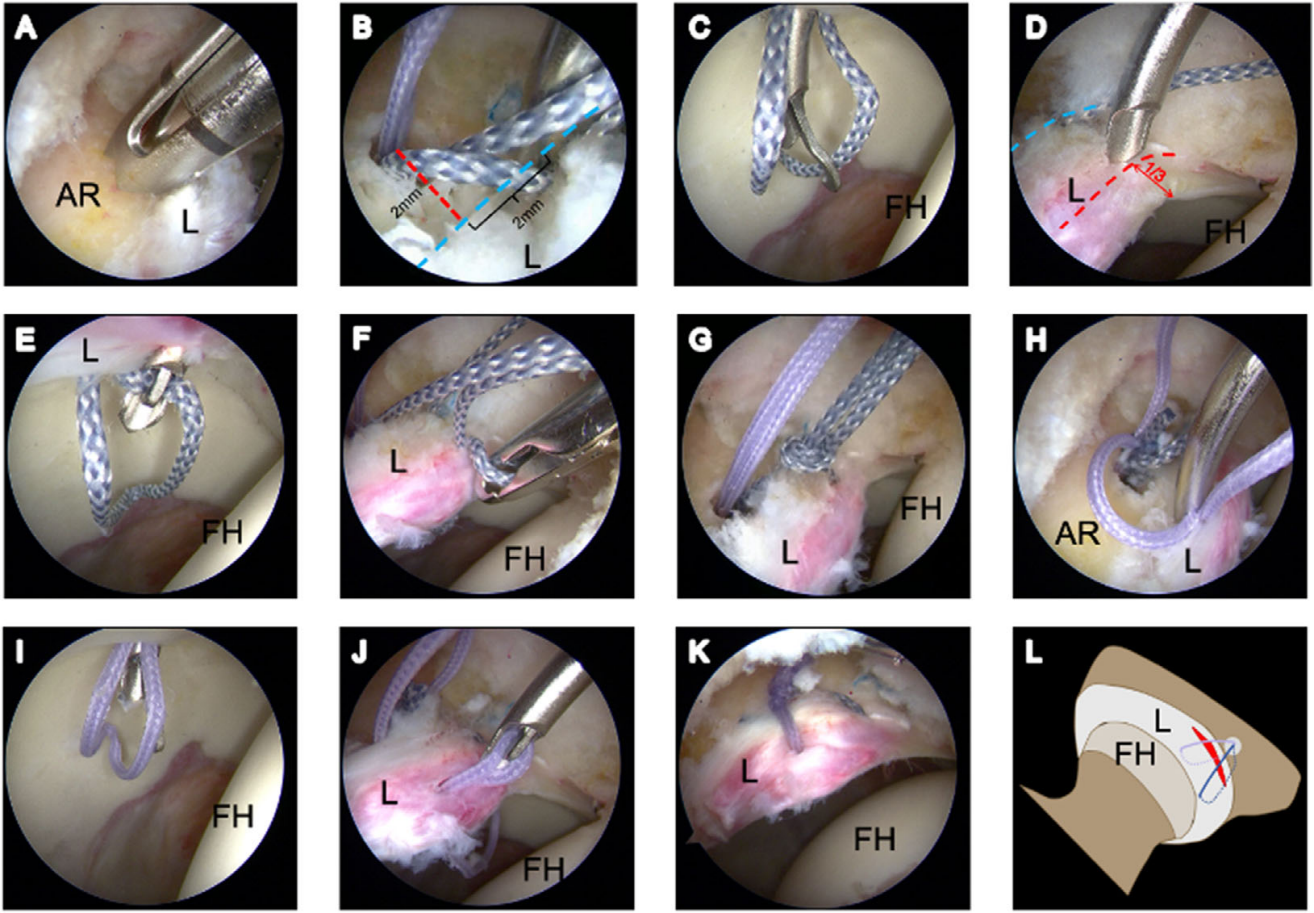
Procedure of inverted V‐shape double‐strand vertical mattress suture technique. Arthroscopic images of a right hip in a supine position, viewing through an anterolateral portal. (A) Double‐strand anchor is placed at the back of the labral injury 1 to 2 mm from the edge of the labrum (the blue line is the boundary of labrum, the red line shows the distance from the anchor to the labrum, and the black bracket shows the suture hook passes through the torn labrum at the labrum and cartilage junction 1 to 2 mm medially from the anchor). (B) One strand is passed through the torn labrum 1 to 2 mm laterally from the anchor corresponding to the injured labrum. (C) The strand enters the central compartment and is released. (D) The suture hook is passed close to the edge of the labrum (the blue line is the boundary of the labrum, the red line shows the outer third of the labrum). (E) The suture is grasped from the central compartment to the back of the labrum. (F) The end of the suture is grasped and withdrawn. (G) Sutures are knotted. (H) The other strand was grasped and displaced 1 to 2 mm laterally from the injured labrum in another side. (I) The suture hook is passed close to the edge of the labrum. (J) The end of the suture is grasped and withdrawn. (K) Sutures are knotted and cut. (L) Schematic diagram of the inverted V‐shape double‐strand vertical mattress suture. (AR, acetabular rim; FH, femoral head; L, labrum.)

Then, labral sutures are performed. Depending on the extent of the injury, a double‐strand anchor (DePuy Synthes, GRYPHON Suture Anchors) is placed in the acetabular bed at the back of the labral injury 1 to 2 mm from the edge of the labrum. The end of 1 strand is grasped with a suture hook. Then, the suture hook passes through the torn labrum at the labrum and cartilage junction 1 to 2 mm medially from the anchor and introduces the suture into the central compartment. The free suture hook is then passed from near the edge of the labrum, approximately at the outer third. The surgeon grasps the end of the suture from the central compartment to the back of the labrum and knots it in place to hold it in position. Then, the other strand is grasped and displaced 1 to 2 mm laterally from the injured labrum on the other side. Similarly, the suture hook passes through the torn labrum and introduces the suture into the central compartment. The suture hook is passed from near the edge of the labrum, and then, the suture is withdrawn, and the surgeon knots it to hold in place. The 2 strands are located on both medial and lateral sides of the anchor point, which is similar to the inverted V‐shape, so it is named the inverted V‐shape double‐strand vertical mattress suture technique and seen in Figure 1.

After the labral suture is completed, traction is relaxed and the hip is flexed at 30° to 45°. A bur is used to grind the cam osteophytes in the head‐neck junction area of the femoral head. Joint flexion and extension movements are examined to determine if there is residual impingement of the hip joint. Video 1 shows the entire surgical technique, including the audio narration.

## DISCUSSION

This article describes a labral suture method that not only preserves the outer edge of the labrum and restores its function but also minimizes the use of anchors, resulting in greater cost‐effectiveness.

Labral repair is recommended when the labral tissue is sufficient and superior at maintaining the seal of joint fluid. In single‐strand loop fixation, the labrum may be bunched, and its normal triangular cross‐sectional anatomy may be distorted.^8^, ^9^, ^10^ The labrum may also be everted away from the femoral head, and thus, the contact of the labrum with the femoral head may not be reproduced.^11^ Therefore, when double strands are used for reinforcement, the compromised basal labrum can cause the strands to be severely bunched, which may impair blood supply and hinder tissue healing, particularly in hypertrophic or difficult‐to‐repair labrums. Single‐strand vertical mattress suture technique can keep the free edge of the labrum intact and avoid eversion of the labrum to reproduce the suction seal by restoring contact with the femoral head.^5^ To preserve the sealing effect and reduce suture damage to the labral parenchyma at the same time, new suture techniques such as the eversion‐inversion labral repair,^12^ inside‐out labral repair,^13^ knotless suture anchor,^14^, ^15^ etc., have been explored and researched.

McGovern et al. have statistically examined the extent of labral tears under arthroscopy and the use of single grand anchors, with the most common tear size being 3 hours (clockface description) and the most common repair location being at the 12 to 3 o'clock position.^16^ Depending on the extent of the labral injury, a varying number of anchors are used. As the efficacy of hip arthroscopy has been proven, its use has increased year after year in the clinic, with the number of hip arthroscopy procedures in the United States increasing by 85% between 2011 and 2018.^17^ However, there are few studies related to the costs of it. Allen et al. retrospectively analyzed the cost of hip arthroscopy for labral injuries and showed that the number of anchors was a significant predictor of increased costs.^5^ In studies of arthroscopic treatment of the shoulder, several studies on the cost of treating rotator cuff tears have drawn similar conclusions.^18^ Maxwell has proposed the use of (No. of knots + No. of tendon suture passes + No. of suture limbs)/No. of pilot holes created to evaluate the efficiency of a rotator cuff suture,^19^ and it is evident that the efficiency of utilization of surgical implants is a key concern for surgeons. In the treatment of labral injuries, we aim to achieve efficacy with a reduced number of anchors. With the use of this technique, the extent of damaged labrum covered by a single anchor is greater than that covered by a conventional single‐strand anchor. In cases of extensive labral injury, or when the patient is economically disadvantaged and needs to save money, this suture technique provides a better clinical option.

When performing an inverted V‐shape double‐strand vertical mattress suture, it is essential to first assess the quality of the labrum, blood supply, and anticipated healing capacity, selecting the best‐quality tissue for suturing. When placing anchors, it is important to plan the anchor positions and spacing in advance based on the extent of the injury. It is recommended to select the distal anterolateral approach approach, which better aligns with the acetabular anteversion, to minimize the risk of anchors penetrating the articular surface or extending into the extra‐articular bone. During suturing, doctors should anticipate the positions of sutures and anchors, and if a suture fails, replan the anchors’ placement accordingly. The limitations of this study are as follows. This technique has high technical requirements and presents a specific learning curve. In addition, biomechanical testing and long‐term clinical follow‐up are needed to validate its long‐term efficacy further. The advantages and disadvantages of this technique are shown in Table 1.

**TABLE 1:** The Advantages and Disadvantages of Inverted V‐Shape Double‐Strand Vertical Mattress Suture Technique

Advantages	Disadvantages
It is possible to use vertical mattress sutures, which can restore the sealing effect	It has high technical requirements and relies on specific equipment, which involves a certain learning curve
It reduces the use of anchors for the same range of injuries, thereby lowering the cost of the procedure and saving on health care costs	There is a lack of biomechanical testing to support it
Its fixation strength is theoretically stronger than that of a single strand, especially in hypertrophied or unstable labrums, which can better restore the anatomical position, ensuring the effectiveness of the surgery	Long‐term clinical follow‐up is needed to verify its long‐term efficacy
If 1 suture fails, the other suture can still function, providing a “double safeguard” for the surgery	

In conclusion, for hip labral injuries, when the labral thickness is sufficient, the inverted V‐shape double‐strand mattress suture technique is an appropriate choice, which is both secure and cost‐effective.

## DISCLOSURES

The authors (Y.H., Q.J., L.H., L.W., M.W., C.L.) declare that they have no known competing financial interests or personal relationships that could have appeared to influence the work reported in this article.
